# Breastfeeding practices and complementary feeding in Ecuador: implications for localized policy applications and promotion of breastfeeding: a pooled analysis

**DOI:** 10.1186/s13006-020-00321-9

**Published:** 2020-08-24

**Authors:** Wilma B. Freire, William F. Waters, Diana Román, Philippe Belmont, Emily Wilkinson-Salamea, Adrián Diaz, Ivan Palacios, Enrique Bucheli

**Affiliations:** 1 Institute for Research in Health and Nutrition, Universidad San Francisco de Quito, Quito, Ecuador; 2 Pan American Health Organization, Quito, Ecuador; 3 School of Medicine, Universidad San Francisco de Quito, Quito, Ecuador; 4 Ministry of Public Health, Quito, Ecuador

**Keywords:** Breastfeeding, Ecuador, Promotion, Policy, Regional analysis

## Abstract

**Background:**

Best practices in breastfeeding are often not followed despite appropriate levels of knowledge and positive attitudes regarding the benefits of human milk. For many reasons, some women do not initiate breastfeeding, suspend breastfeeding early, or initiate complementary feeding earlier than recommended. Usual measurement methods use large sample surveys at a national scale, which are not well suited for monitoring sub-national differences.

**Methods:**

In order to understand how local infant feeding practices could influence policy and promotion practices, we apply data pooling methodology to analyse breastfeeding patterns in different Ecuadorian settings: Cumbayá parish, located near Quito, the Ecuadorian capital; the city of Macas and rural surroundings in the Amazon basin province of Morona Santiago; and the province of Galapagos. Surveys were conducted independently between August 2017 and August 2018; while they are representative of each respective setting, sampling designs and survey methods differ, but the same demographic information and data based on standard breastfeeding indicators established by the World Health Organization (WHO) were collected. In order to account for differences in the different settings, the design effect of each survey was considered in the analysis.

**Results:**

Significant differences were found in breastfeeding practices between the suburban Cumbayá parish near Quito and Galapagos on one hand, and urban and rural parts of Morona Santiago, on the other. The rates of early breastfeeding initiation and age-appropriate breastfeeding are significantly higher in urban and rural Morona Santiago then in Cumbayá or Galapagos, while the rate of exclusive breastfeeding is highest in rural parts of Morona Santiago. No significant differences were found in complementary feeding practices between Cumbayá and Galapagos, but there are with urban and rural Morona Santiago. Initiation of breastfeeding in the first hour after birth occurs in only 36.2% of cases in Cumbayá but in 75.4% of cases in urban Morona.

**Conclusions:**

Differences among regions reflect specific opportunities and barriers to practices related to promoting optimal infant health and nutrition. Consequently, regional or local conditions that often are not apparent in national-level data should orient policies and promotion activities in specific populations.

## Background

Many mothers throughout the world do not breastfeed, suspend breastfeeding early, or initiate complementary feeding earlier than recommended by international organizations [1]. The World Health Organization (WHO) recommends that all newborns initiate breastfeeding in the first hour after birth and continue exclusive breastfeeding (EBF) for six months and complementary feeding for an additional 18 months or more. The benefits of breastfeeding and correct timing of EBF and complementary feeding are well established [1]. Human milk is a nutritious and safe food that is easily digested and absorbed, and provides appropriate levels of vitamins, minerals, fat, proteins, and energy. Health benefits that accrue to infants include improved nutritional status and survival rates, prevention of infectious disease in infancy and of chronic disease (including diabetes), and obesity in adulthood [2]. Breastfeeding also provides for extension of post-pregnancy amenorrhea [3]. In social terms, one of the greatest benefits is that breastfeeding is free and safe, which are critical advantages in poor populations [4, 5]. Most mothers can and should breastfeed; only in exceptional circumstances is breastmilk contraindicated [6].

Despite these many benefits, reported rates of breastfeeding are generally lower than would be expected. The proportion of infants from birth to five months of age who are exclusively breastfed is only 42% worldwide, 38% in Latin America and the Caribbean, and 39.6% in Ecuador, as compared to 58.3% in Bolivia, 66.4% in Peru, 36.1% in Colombia, and 32.0% in Argentina [7]. Dramatic as these data are, however, national-level data obfuscate important regional differences, which remain to be fully elucidated. The analysis of breastfeeding practices at the national level requires large amounts of data that are not well suited for evaluation at the subnational level and the lack standardization methods limits the validity of dataset comparisons [8].

Factors that contextualize less-than-optimal breastfeeding, EBF and complementary feeding practices throughout the world include maternal age, low educational level, low income, urban residence, institutionalized healthcare that does not conform to baby-friendly norms, perceived insufficiency of breast milk supply, maternal or infant illness, discomfort or injury, previous inability to breastfeed, lack of social support, emotional stress, and the pressure of advertising that touts purported advantages of industrialized milk substitutes [4, 9, 10]. Maternal employment represents an important barrier to appropriate breastfeeding practices when women are obliged (often for economic reasons) to return to work without appropriate conditions for continuation of breastfeeding [11]. We describe some of these contextual factors in this paper, whose aim is to show how the analysis of pooled data from independent subnational surveys in different Ecuadorian settings can influence breastfeeding policy and promotion practices at the regional or local level. This methodology can be relevant in other parts of the region and the world.

The Quito and Galapagos surveys were approved by the IRB of the Universidad San Francisco de Quito, while the Morona Santiago survey was approved by the IRB of the Universidad de las Americas, both in Quito, Ecuador.

## Methods

This paper analyses breastfeeding patterns in different settings in Ecuador, where the diverse geographical, socio-economic, and cultural milieu strongly influences local health and nutrition related behaviour. While the variables discussed here may be specific to Ecuador, a similar analysis can be applied elsewhere. This study was designed to understand the degree to which breastfeeding indicators, which are generally reported at the national level, may limit the ability to obtain accurate estimators for specific age groups and to translate those data into locally appropriate breastfeeding promotion and policies that reflect social, economic, and cultural specificities.

### Study sites

Located in Ecuador’s northern highlands, Cumbayá parish is home to a heterogeneous population of long-time residents who maintain rural lifestyles alongside newer, often wealthier residents, many of whom commute 10 km to Quito, the nation’s capital. According to the most recent census, Cumbayá had 31,463 residents in 2010 compared to a total of 21,078 in the previous (2001) census [12], representing a 10-year growth rate of 33%. Although Cumbayá is classified as a rural parish, due to its close geographical, economic, and social proximity to Quito and the rapid development of office buildings, shopping centres, and residential clusters referred to as *urbanizaciones*, Cumbayá in many respects resembles North American and European suburbs in terms of access to goods and services, including healthcare. In 2010, 3.4% of Cumbayá’s residents identified themselves as indigenous, compared to the national figure of 7.0% [13]. In summary, this parish is similar to other places characterized by relatively rapid social change and economic development and where access to healthcare is favourable.

The province of Morona Santiago, located in the southern part of Ecuador’s tropical Amazon region, had a population of 147,940 in 2010; one third lived in urban areas (mostly in the provincial capital of Macas) and two thirds in rural areas. Nearly half (48.4%) of the province’s residents identified themselves as indigenous [13]. This province is similar to other parts of the country where social change and economic development have proceeded more slowly and where access to healthcare and other services is limited.

The province of Galapagos is renowned for its endogenous animal and plant species but was also home to 25,124 residents in 2010 and 25,244 in 2015. In all, 82.5% of residents live in urban areas, mostly in the two largest cities of Puerto Ayora and San Cristobal. Before regulations were promulgated to limit permanent settlement, immigration was rapid, especially from the coastal mainland. Although a community of highland indigenous residents also developed, such that 7.0% of *Galapageños* identified themselves as indigenous in 2010 [13, 14]. Galapagos is similar to other tropical parts of Ecuador in terms of climate, but is also a place of more rapid, specialized development because of its status as a global tourist attraction. At the same time, while the urban population has access to basic public and private healthcare, the province is isolated from the rest of the country and lacks specialized healthcare services.

### Data and collection and management

Surveys were conducted independently in Cumbayá, urban and rural Morona Santiago, and Galapagos between August 2017 and August 2018. Data collection was conducted by different research groups, and datasets were subsequently standardized and merged through data pooling for this analysis. While different sampling methods were employed, each dataset was representative of the respective subregion. The surveys included mothers of infants between 0 and 59 months of age, who did not suffer from acute or chronic illnesses, and who provided informed consent. The questionnaire was adopted from an instrument designed by WHO [15, 16], which collected information on household composition and indicators to assess infant feeding practices for evaluating breastfeeding practices worldwide. Socio-economic information and birthing history included in the questionnaire provided data on informants’ age, marital status, employment, educational level, number of childbirths, and type of delivery.

Our analysis was conducted using indicators established by WHO [15, 16], which we divide into four groups. The first group of two indicators are linked because the probability of age-appropriate breastfeeding practices is closely associated with successful early initiation.

1. *Early initiation of breastfeeding*: percentage of children born in the past 24 months who were put to the breast within one hour of birth.
2. *Age-appropriate breastfeeding*: percentage of infants 0–5 months of age who receive only breast milk and of children 6–23 months of age who received breast milk as well as solid, semi-solid, or soft foods during the previous day.

The second group of five indicators reflect appropriate breastfeeding practices during different stages of infancy and early childhood.

3. *Exclusive breastfeeding (< 6 months)*: percentage of infants 0–5 months of age who were fed exclusively with breastmilk during the previous day.
4. *Continued breastfeeding:* proportion of children 12–15 months who are fed breast milk.
5. *Infants ever breastfed*: percentage of children born in the past 24 months who were ever breastfed.
6. *Continued breastfeeding to 24 months*: percentage of children 20–23 months of age who are fed breast milk.
7. *Predominant breastfeeding (< 6 months)*: percentage of infants 0–5 months of age previous day.

The third group is composed of five indicators that reflect different aspects of complementary feeding.

8. *Introduction of solid, semi-solid, or soft foods (6–8 months)*: percentage of infants 68 months of age who were fed with solid, semi-solid, or soft foods during the previous day.
9. *Minimum dietary diversity (6–23 months*): percentage of children 6–23 months of age who received foods from at least 5 out of 8 defined food groups during the previous day.
10. *Minimum meal frequency (6–23 months)*: percentage of children 6–23 months of age who received solid, semi-solid, or soft foods (but also including milk feeds for non-breastfed children) the minimum number of times or more during the previous day.
11. *Minimum acceptable diet (6–23 months)*: percentage of children 6–23 months of age who received a minimum acceptable diet during the previous day.
12. *Bottle feeding (0–23 months)*: proportion of children 0–23 months of age who were fed with a bottle during the previous day.

The final indicator refers to children who were not breastfed.

13. *Milk feeding frequency for non-breastfed children*: proportion of non-breastfed children 6–23 months of age who received a least two milk feedings during the previous day

The questionnaire was applied in Spanish in Cumbayá and Galapagos, while in some cases, it was applied in indigenous languages in Morona Santiago after validation by trained bilingual interviewers. Sample sizes were calculated considering a standard normal deviation of 1.96, adjusted by expected prevalence of appropriate breastfeeding prevalence for children 0 to 24 months of age in each subregion: 0.5 in Galapagos and 0.48 in Cumbayá and Morona Santiago, using calculations from the national health and nutrition survey [17] and a 5% margin of error [18]. Additional adjustments for finite population size and non-response rates of 5% in urban areas and 2% in rural areas were applied for each subregion [19]. In the case of Galapagos, children above 24 months were not part of study. See Table 1.

**Table 1:** Sample size by study site and infants’ age groups

	Age group (months)	Study site			
		(Cumbayá)	Galapagos	Morona Santiago (urban)	Morona Santiago (rural)
Age groups	0 to 5	81	51	57	106
	6 to 23	147	187	142	237
	24 to 59	58	0	275	486
	Total:	286	238	474	829

Trained personnel conducted face-to-face interviews with eligible mothers in Cumbayá, urban and rural Morona Santiago, and Galapagos. Participants in Cumbayá were recruited opportunistically, identified, and selected through random sampling among women who attended either of two public health centres. Participation was solicited when women entered the health centres and a description of the study was provided along with the informed consent procedure. This process continued until the calculated sample size was reached.

In Morona Santiago, a representative sample of rural and urban residents was employed using national census tract definitions. As in Cumbayá, participants were recruited for voluntary participation and informed consent upon entering public health centres.

In Galapagos, a preliminary list of children from 0 to 24 months of age was obtained from government-operated day care centres. To reach the required number of children, snowball sampling was applied to obtain additional participants in order to arrive at the required sample size. A total of 279 women were surveyed and 247 valid interviews were included in the analysis.

### Data analysis

Indicators for feeding practices are reported using weighted data and calculated using the Demographic and Health Survey (DHS) approach to handling missing data [10]. Data cleaning and post-stratification were performed for the Galapagos and Morona surveys using additional demographic data and a ranking algorithm. The Cumbayá dataset required no adjustment after the field survey. In order to calculate differences in indicators between Cumbayá, Galapagos, and urban and rural Morona Santiago, the Tukey Contrast test was used to assess multiple comparisons of means (MCT) for each pair of surveys in order to determine which means among a set of means differ from the others [20]. The test compares the difference between each pair of means with appropriate adjustments for multiple testing to account for bias between surveys.

## Results

This section provides an analysis of breastfeeding practices as measured by the 12 breastfeeding indicators discussed above. First, Table 2 provides descriptive data on socio-economic characteristics in the study locations in order to contextualize factors that could impact regionally appropriate promotion and policy actions. It can be seen that most women in the four study sites were married and lived with a partner, although the proportion was lower in urban (U) and rural (R) Morona Santiago, where the proportion of single mothers was higher. In Cumbayá, over half of women reported having a university education while in Galapagos, nearly a third reported a university education and over half had a high school education. In contrast, less than half of respondents in urban and rural Morona Santiago had attended a university and a high proportion reported a primary school education.

**Table 2:** Sample description of mothers’ socio-economic characteristics

Indicators		Cumbayá		Galapagos		Morona Santiago (urban)		Morona Santiago (rural)	
		*n*	%	*n*	%	*n*	%	*n*	%
Marital status	Divorced or separated	11	4	15	6	13	4	33	7
	Married with partner	257	85	207	85	240	78	389	79
	Single	33	11	22	9	55	18	73	15
Educational level	Primary	21	7	25	10	125	40	268	54
	Secondary	108	36	143	58	164	53	217	44
	University	172	57	78	32	20	6	10	2
Mother’s age	15 to 19	9	3	22	9	58	19	76	15
	20 to 29	123	41	114	46	159	52	256	52
	30 to 49	169	56	111	45	92	30	162	33
Type of birth	Cesarean	175	58	97	39	13	4	7	1
	Vaginal	126	42	149	61	296	96	488	99
	Total	301	100	247	100	309	100	495	100

In Cumbayá, the largest proportion of mothers was between 30 and 39 years of age, while in Galapagos, more than 90% were almost equally divided between the 20–29- and 30–39-years age groups. In contrast, a higher proportion of mothers in urban and rural Morona Santiago were between 15 and 19 years of age. With respect to type of birth, Cumbayá and Galapagos differ from Morona Santiago in that the proportion of women reporting Caesarean sections was high compared to WHO guidelines [20], while in the latter, few women had delivered via Caesarean section.

Table 3 presents comparisons of infant feeding indicators for the four study locations. For each indicator, Tukey Contrasts assess multiple comparisons of means for each pair of surveys, or all pairwise comparisons using the R *multcomp* package [21]. They report statistically significant differences (*p* < 0.5). For readability, comparable means are reported with subscript letters ‘a’ through ‘e’. The absence of subscript indicates that a reported mean is statistically different from all other means. In Galapagos, children for those indicators include those between 0 and 24 months of age.

**Table 3:** Multiple mean Comparison of WHO Breastfeeding practices indices, between the four study areas

Indicator	Study site	***n***	%	SD
A.				
1. Early initiation of breastfeeding.	Cumbaya	296	36.2	0.48
	Galapagos	246	62.5	0.48
	Morona U	277	75.4^a^	0.43
	Morona R	449	74.7^a^	0.43
2. Age-appropriate breastfeeding (0–59 months)*	Cumbaya	301	47.8	0.50
	Galapagos	247	62.0	0.49
	Morona U	475	20.1^a^	0.40
	Morona R	834	17.6^a^	0.38
B.				
3. Exclusive breastfeeding to 6 months	Cumbaya	81	51.9^ab^	0.50
	Galapagos	51	55.9^ac^	0.50
	Morona U	52	56.7^bcd^	0.50
	Morona R	96	84.0^d^	0.37
4. Continued breastfeeding, 12–15 months	Cumbaya	43	76.7^ab^	0.43
	Galapagos	56	82.4^ac^	0.38
	Morona U	32	56.5^cd^	0.50
	Morona R	63	52.4^d^	0.50
5. Infants ever breastfed.	Cumbaya	301	98.4^a^	0.13
	Galapagos	247	99.5^a^	0.07
	Morona U	309	93.0^b^	0.26
	Morona R	495	90.8^b^	0.29
6. Continued breastfeeding at 2 years.	Cumbaya	29	24.1^abc^	0.44
	Galapagos	38	41.1^ade^	0.49
	Morona U	27	13.8^bdf^	0.35
	Morona R	56	33.8^cef^	0.47
7. Infants < 6 months predominantly breastfed.	Cumbaya	80	61.3^ab^	0.49
	Galapagos	51	65.5^acd^	0.48
	Morona U	52	69.7^bce^	0.46
	Morona R	96	84.0^de^	0.37
C.				
8. Infants who received solid, semi-solid, or soft foods	Cumbaya	37	97.3^a^	0.16
	Galapagos	37	92.0^a^	0.27
	Morona U	29	70.6	0.46
	Morona R	44	43.2	0.50
9. Infants 6–23 months with minimum dietary diversity.	Cumbaya	162	88.3^a^	0.32
	Galapagos	196	89.6^a^	0.31
	Morona U	154	57.2	0.50
	Morona R	255	32.4	0.47
10. Infants 6–23 months with minimum meal frequency.	Cumbaya	162	87.7^a^	0.33
	Galapagos	196	90.5^a^	0.29
	Morona U	148	52.1	0.50
	Morona R	250	20.6	0.40
11. Infants 6–23 months with minimum acceptable diet.	Cumbaya	162	72.2^a^	0.45
	Galapagos	196	74.5^a^	0.44
	Morona U	149	23.9	0.43
	Morona R	252	8.8	0.28
12. Bottle feeding. Proportion of children 0–23 months of age who are fed with a bottle.	Cumbaya	242	46.7^a^	0.50
	Galapagos	246	40.8^a^	0.49
	Morona U	211	28.0	0.45
	Morona R	361	12.8	0.33
D				
12. Milk feeding frequency for non-breastfed. Proportion of non-breastfed children 6–23 months of age who receive at least 2 milk feedings.	Cumbaya	59	79.7	0.41
	Galapagos	63	79.5^a^	0.40
	Morona U	–	–	–
	Morona R	–	–	–

* 0–25 months in Galapagos

In Panel A of Table 3, it can be seen that the rates of early breastfeeding initiation and age-appropriate breastfeeding are significantly higher in urban and rural Morona Santiago then in Cumbayá or Galapagos.

The data presented in Panel B of Table 3 suggest that EBF and complementary feeding practices do not differ significantly among the four study locations. In general, these data reflect a relatively high rate of EBF for six months, while breastfeeding rates are reduced thereafter until two years of age. It can be seen, though, that the EBF rate is higher in rural parts of Morona Santiago, reflecting both the persistence of cultural beliefs of largely indigenous communities as well as limited resources and access to industrialized milk substitutes. Interestingly, though, rates of continued breastfeeding are lower in urban and rural Morona Santiago than in their more urbanized counterparts. The comparisons of predominant breastfeeding under six months (indicator 7) reveal that proportionately, more than half of infants < 6 months are breastfed, and that again, the practice is most evident in rural Morona Santiago.

Panel C of Table 3 reports on data related to complementary feeding practices. It can be seen that there are no significant differences between Cumbayá and Galapagos, but that there are with urban and rural Morona Santiago, reflecting less appropriate practices in the latter.

Finally, Panel D of Table 3 reports on milk feeding frequency for non-breastfed infants from 6 to 23 months of age. These data, collected only in Cumbayá and Galapagos, show no significant differences between those two study sites, in that in both places, a large proportion of infants received at least two portions of milk the day before the survey.

## Discussion

Achieving broad compliance with optimal breastfeeding practices has proved to be elusive. While national-level data provide interesting snapshots and allow for international comparisons, they are less useful for developing and implementing effective policies and promotion strategies that can make a difference at the regional and local level in communities with characteristics that may inhibit successful linkages between knowledge, attitudes, and practices. It is not surprising, then, that the way in which different groups of women in the study areas manage EBF and complementary feeding do not necessarily conform to recommendations established by international organizations [1, 6]. Nor do they necessarily reflect levels of knowledge and attitudes with regard to the benefits of breastfeeding.

Differences among specific population segments reflect a variety of opportunities and barriers to best practice in promoting optimal infant health and nutrition [22]. As would be the case elsewhere in the world, a consideration of contextual sociodemographic conditions suggests that in comparing women in the four study sites, those in Cumbayá are somewhat older on average, have higher level of education, are mostly non-indigenous, and are more likely to have given birth via Caesarean section. In contrast, those in Morona Santiago are on average younger, have lower levels of education, are much more likely to be indigenous, and to have not undergone Caesarean sections. The proportions of these indicators are intermediate in Galapagos. These factors, and others that are locally relevant, provide insights as to why pooled data from independent surveys reveal significant differences. Additionally, early initiation of breastfeeding (within the first hour) and age-appropriate breastfeeding practices may be related to the persistence of cultural practices, in this case, among indigenous residents of Morona Santiago. Although indigenous populations are not heterogeneous and breastfeeding practices may be declining, they may still be protected by the lower rates of Caesareans although complementary feeding practices may be less than optimal due to poorer socio-economic conditions [23–25]. Conversely, while women in Cumbayá and Galapagos have more advantageous access to health services in general, they are more likely to give birth through Caesarean sections and in addition, to have greater access to milk substitutes and to mass-media advertising.

While this study emphasizes an approach to incorporating regional and local data into policy and promotion decision making, several limitations must be acknowledged. First, like any case study, the degree to which findings can be extrapolated to other places and other situations is an issue. In that regard, the data provided in this paper demonstrate differences that may be similar to other places where geographic location, rurality, and relative isolation are important factors. Similarly, it is always important to contextualize sociodemographic conditions. In this case, indigenous identity is one such factor. We do not perform a statistical analysis of sociodemographic factors because the focus of this paper is not on determinants but on the pooling of data from three independent regional surveys which, we suggest, can be a useful tool that can be implemented elsewhere.

Second, as discussed earlier, the use of data derived from independent surveys implies differences in sampling strategies and outcomes. Nevertheless, the independent samples are all representative of the respective populations and having been pooled, permit comparison.

## Conclusions

This paper argues that an understanding of regional and local breastfeeding practices may be obscured by national level data, and that appropriate analytical approaches can be used to elucidate relevant subnational factors. Most importantly, developing appropriate and effective policy and promotion strategies can be based on relevant factors such as early initiation of breastfeeding, age appropriate breastfeeding practices, and rates of Caesarean sections in different parts of a given country. For example, the proportion of infants who benefitted from timely initiation of breastfeeding is quite high in Morona Santiago but surprisingly low in Cumbayá, where mothers can more easily receive prenatal care and give birth in well-equipped private hospitals as well as in public facilities. Ready access to quality healthcare is clearly advantageous but at the same time, it is contradictory that the rates of Caesarean sections are high in Cumbayá, representing a potential barrier to early initiation of breastfeeding [26], which in turn can affect other age-appropriate breastfeeding practices. Conversely, complementary feeding practices are less adequate in Morona Santiago, where rural mothers, many of them very young, are likely to be poor and hence, may not have access to nutritious complementary foods or to appropriate accurate nutrition and health information [27].

Established WHO breastfeeding indicators are widely used in a variety of settings, but they are not sensitive enough to provide information on the variability of breastfeeding practices at regional or local level. Therefore, it is necessary to include factors at those levels in order to better understand knowledge, attitudes, and practices that are fundamental to early initiation of breastfeeding and the transition from to complementary feeding, when it is essential to introduce appropriate foods that not only provide adequate nutrients, but also generate healthy eating practices that will last throughout the life cycle, since even moderately poor nutrition during infancy can lead to irreversible nutritional problems in the long term [28, 29]. In that context, on one hand, feeding practices during infancy and early childhood are critical precursors to good health and nutrition throughout the life cycle, so that promotion of appropriate complementary feeding strategies is essential [19, 30], taking into account locally- and regionally specific factors such as income and level of mothers’ education [31–33]. In that regard, behavioural sciences can contribute significantly to promoting positive change in nutritional outcomes using, for example, innovative tools such as social media platforms to promote appropriate breastfeeding behaviours [34].

Additionally, a broad range of policy options is available, including the provision of appropriate services provided by personnel trained in breastfeeding protection and promotion in baby-friendly hospitals, community outreach, and the control of the inappropriate distribution and promotion of milk substitutes [35] because optimal breastfeeding practices are undermined in many parts of the world by the early introduction of industrialized milk substitutes to the detriment of newborns’ health and wellbeing. A study of the violations of the International Code of Marketing of Breast Milk Substitutes reports that many mothers of newborn babies and infants receive promotional materials, free samples, diapers, and bottles to mothers of newborns and infants from representatives of milk substitute producers and that in many cases, paediatricians recommended the use of milk substitutes [27].

## Data Availability

The datasets used and/or analysed during the current study are available from the corresponding author upon reasonable request.
